# Leonurine Ameliorates Oxidative Stress and Insufficient Angiogenesis by Regulating the PI3K/Akt-eNOS Signaling Pathway in H_2_O_2_-Induced HUVECs

**DOI:** 10.1155/2021/9919466

**Published:** 2021-08-03

**Authors:** Li Liao, Lihong Gong, Mengting Zhou, Xinyan Xue, Yunxia Li, Cheng Peng

**Affiliations:** ^1^State Key Laboratory of Southwestern Chinese Medicine Resources, Chengdu 611137, China; ^2^School of Pharmacy, Chengdu University of Traditional Chinese Medicine, Chengdu 611137, China; ^3^National Key Laboratory Breeding Base of Systematic Research, Development and Utilization of Chinese Medicine Resources, Chengdu 611137, China

## Abstract

Thrombus is considered to be the pathological source of morbidity and mortality of cardiovascular disease and thrombotic complications, while oxidative stress is regarded as an important factor in vascular endothelial injury and thrombus formation. Therefore, antioxidative stress and maintaining the normal function of vascular endothelial cells are greatly significant in regulating vascular tension and maintaining a nonthrombotic environment. Leonurine (LEO) is a unique alkaloid isolated from *Leonurus japonicus* Houtt (a traditional Chinese medicine (TCM)), which has shown a good effect on promoting blood circulation and removing blood stasis. In this study, we explored the protective effect and action mechanism of LEO on human umbilical vein endothelial cells (HUVECs) after damage by hydrogen peroxide (H_2_O_2_). The protective effects of LEO on H_2_O_2_-induced HUVECs were determined by measuring the cell viability, cell migration, tube formation, and oxidative biomarkers. The underlying mechanism of antioxidation of LEO was investigated by RT-qPCR and western blotting. Our results showed that LEO treatment promoted cell viability; remarkably downregulated the intracellular generation of reactive oxygen species (ROS), malondialdehyde (MDA) production, and lactate dehydrogenase (LDH); and upregulated the nitric oxide (NO) and superoxide dismutase (SOD) activity in H_2_O_2_-induced HUVECs. At the same time, LEO treatment significantly promoted the phosphorylation level of angiogenic protein PI3K, Akt, and eNOS and the expression level of survival factor Bcl2 and decreased the expression level of death factor Bax and caspase3. In conclusion, our findings suggested that LEO can ameliorate the oxidative stress damage and insufficient angiogenesis of HUVECs induced by H_2_O_2_ through activating the PI3K/Akt-eNOS signaling pathway.

## 1. Introduction

As a chronic multifactorial disease, thrombosis refers to blood clots forming in arteries or veins. It is considered the pathological phenomenon of cardiovascular disease and thrombotic complications as it often causes myocardial infarction, ischemic stroke, coronary heart disease, acute atherosclerotic syndrome, and pulmonary embolism. Additionally, the death and prognosis of the current pandemic caused by SARS-CoV-2 (the aetiological agent of COVID-19) were proved to be related to thrombosis [1]. Therefore, the thrombus is seriously threatening people's life and health [2, 3].

Vascular endothelial cells, the critical regulator to maintain vascular health and normal function, together with platelets and circulating coagulation proteins, are crucial mediators of thrombosis. Vascular endothelial cells are considered to be the center of vascular diseases as they have anticoagulant, antithrombotic, and plasminogen properties and play an indispensable role in regulating vascular tension and maintaining homeostasis [4]. Vascular endothelial cell injury may expose fibrinogen, induce monocyte/macrophage aggregation and adhesion, promote coagulation and platelet aggregation [5], also increase the release of ET-1 and platelet IV factor, and reduce the release of PGI_2_ and NO [6], thus inducing or accelerating the formation of thrombosis. At present, many antithrombotic drugs can significantly reduce cardiovascular adverse events. However, the curative effect and prognosis are still limited due to varying degrees of adverse reactions, such as bleeding, liver and kidney dysfunction, or stomachache [7]. Therefore, the research and development of effective drugs to protect endothelial cells have an extensive prospect in preventing and treating thrombotic diseases.

Oxidative stress refers to excessive production of high activity enzymes such as living nitrogen free radicals or reactive oxygen species (ROS), which leads to the imbalance of cellular antioxidant capacity. Substantial evidence suggested that oxidative damage was crucial in both vascular endothelial cell injury and insufficient angiogenesis in the process of tissue repair, which may lead to aggravating thrombosis [4]. As a kind of reactive oxygen species (ROS) produced by the body, H_2_O_2_ produces a large amount of ROS at high concentrations, which may cause oxidative damage to endothelial cells [8, 9]. Therefore, H_2_O_2_ is widely used to induce oxidative damage and replicate the apoptosis model [10].

*Leonurus japonicus* Houtt is a traditional herb, which has a significant effect on promoting blood circulation and removing blood stasis syndrome. It has been widely used in the treatment of blood stasis syndrome for thousands of years. LEO is a unique alkaloid isolated from *Leonurus japonicus*. *Leonurus japonicus* Houtt is a common medicine of TCM for promoting blood circulation, resolving stasis, and regulating menstruation, diuresis, and detumescence. It has been widely used for centuries to treat dysmenorrhea, menstrual disorders, and other gynecological diseases [11]. LEO is a specific alkaloid only found in *Leonurus japonicus* Houtt. Modern pharmacological studies have shown that it has a variety of biological activities such as vasodilation [12], antiplatelet aggregation and inhibition of vasoconstriction [13–16], anticoagulant [17], anti-inflammatory [18], antioxidative [19], anti-ischemia, antiapoptosis [20, 21], and heart protection [22]. However, whether it can repair vascular endothelial cell injury and promote angiogenesis through antioxidant stress has not been clarified.

In this study, we established an H_2_O_2_-induced oxidative injury model of HUVECs and explored the effects of LEO on the repairing and angiogenesis after oxidative stress injury. Our results showed that LEO protected HUVECs from H_2_O_2_-induced endothelial dysfunction by improving the oxidative stress index (ROS, LDH, MDA, and SOD) and cell apoptosis. Besides, LEO potently stimulated eNOS activation and endothelial NO production by activating the PI3K/Akt-eNOS signaling pathway, which may benefit antithrombosis.

## 2. Materials and Methods

### 2.1. Materials

**Figure 1:**
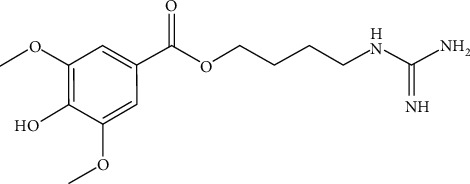
Chemical structure of LEO.

LEO (purity ≥ 98%, the chemical structure of LEO is shown in Figure 1) was purchased from the Must Bio-Technology Company, China. 1640 medium and fetal bovine serum (FBS) were purchased from Gibco (Australia). Trypsin (1 : 250) was purchased from BIOFROXX (Guangzhou, China). Human umbilical vein endothelial cells (HUVECs) were obtained from the School of Pharmacy, Chengdu University of Traditional Chinese Medicine (Sichuan, China). MTT was from Biosharp (Beijing, China). Matrigel Basement Membrane Matrix was purchased from Corning (New York, USA). Nitric oxide (NO), malondialdehyde (MDA), lactate dehydrogenase (LDH), and superoxide dismutase (SOD) commercial kits were obtained from Elabscience Biotechnology Co., Ltd (Wuhan, China). 2,7-Dichlorofluorescein diacetate (DCFH-DA) was purchased from Yeasen Biotechnology Co., Ltd. (Shanghai, China). The BCA protein assay kit, phenylmethylsulfonyl fluoride (PMSF), and RIPA lysis buffer were purchased from Beyotime (Jiangsu, China). The Cell Total RNA Isolation Kit was purchased from Foregene Biotechnology Co., Ltd (Chengdu, China); 5x All-In-One MasterMix and Eva Green 2x RT-qPCR MasterMix-Low RoX were purchased from Applied Biological Materials Inc. (Richmond, BC, Canada). Specific rabbit polyclonal antibodies to endothelial nitric oxide synthase (eNOS), phospho-eNOS (Ser1177), PI3K p85, phospho-PI3K (Tyr607), Akt1/2/3, phospho-Akt1/2/3 (Ser473), Bax, Bcl2, GAPDH, and *β*-actin (as a loading control) were purchased from Affinity Biosciences. Other chemicals and reagents used in this study were obtained from Kelong Chemical Reagent Factory (Chengdu, China).

### 2.2. Cell Culture

HUVECs were incubated at 37°C with 95% humidity and 5% CO_2_. Unless otherwise indicated below, cells were maintained in 1640 medium supplemented with 100 U/ml penicillin, 100 U/ml streptomycin, and 10% fetal bovine serum (FBS) at 37°C, 5% CO_2_.

### 2.3. Determination of LEO Concentration

The MTT cell assay was taken to study the cytotoxic effect of LEO on HUVECs. Cells were seeded in 96-well plates at a density of 8 × 10^3^ cells per well, 100 *μ*l per well with 10% FBS culture medium. After 24 h incubation, cells were treated with different concentrations of LEO (0-1000 *μ*M) that dissolve in DMSO and dilute with 1640 medium containing 1% FBS. The control group was treated only with the 1640 medium containing 1% FBS at 37°C in a 5% CO_2_ incubator for 24 h. Then, MTT solution (20 *μ*l) was added to each group, and cells were incubated at 37°C for another 4 h. After that, the MTT solution was discarded. Cells were then dissolved by adding DMSO (150 *μ*l per well), and the solutions were mixed thoroughly for 5 min. Finally, the absorbance was determined at 570 nm with a BIO-RAD microplate reader (Benchmark Plus, USA). The absorbance of untreated cells was regarded as 100% of cell survival. Cell viability = (treated viable cells)/(control viable cells) × 100%.

### 2.4. Oxidative Stress Injury Model by H_2_O_2_

HUVECs (8 × 10^3^ cells per well) were inoculated in a 96-well plate and cultured for 24 h. HUVECs were exposed to (0-1200 *μ*M) H_2_O_2_ for 24 h, and then, cell viability was measured by the MTT assay as described in Section 2.3.

### 2.5. Cell Viability Assay

HUVECs (8 × 10^3^ cells per well) were inoculated in a 96-well plate and cultured for 24 h. HUVECs were exposed to (2.5, 5, and 10 *μ*M) LEO with H_2_O_2_ (200 *μ*M) for 24 h. The cell viability was measured by the MTT assay as described in Section 2.3.

### 2.6. Cell Morphological Observation

To observe the effect of drugs on cell morphology, HUVECs (2 × 10^5^ cells per well) were plated in 6-well plates and treated with different concentrations of LEO (2.5, 5, and 10 *μ*M) and H_2_O_2_ (200 *μ*M) for 24 h after incubation at 37°C for 24 h. Then, the morphology of the cells was observed and photographed by an inversion fluorescence microscope (Leica DMI3000B, Germany).

### 2.7. Cell Migration

HUVECs (2 × 10^5^ cells per well) were seeded in 6-well plates. After attachment, a rectangular wound was gently and slowly scratched in the center of the cell monolayer using a 200 *μ*l sterile plastic pipette tip. The wounded monolayer was rinsed with PBS and then incubated with basal medium containing 1% FBS with various concentrations of LEO (2.5, 5, and 10 *μ*M) for 12 h. Images of the wounds were recorded in five random fields (×100) with a phase-contrast microscope (Leica DMI3000 B, Germany) at 0 h and 12 h. The scratch areas were measured using ImageJ. The wound healing ability was quantified by the formula as follows:

(1) $$\begin{array}{c} \text{Wound closure}\%=\frac{\text{wound areas on }0\text{ h}-\text{wound areas on }12\text{ h}}{\text{wound areas on }0\text{ h}}\times 100\%. \end{array}$$

### 2.8. Tube Formation

As a result of the migration of HUVECs, the formation of new blood vessels can bring new blood flow to improve local ischemic necrosis and repair damaged tissue [23, 24]. To further study the pharmacological effect of LEO on angiogenesis, a tube forming experiment was performed to observe the effect of LEO on angioplasty. The Matrigel Basement Membrane Matrix was thawed at 4°C, then added to a 96-well plate (20 *μ*l per well) and polymerized at 37°C for 0.5 h. HUVECs (2 × 10^5^ cells per well) were seeded into the coated plate containing 1% FBS with LEO (2.5, 5, and 10 *μ*M) and H_2_O_2_ (200 *μ*M), H_2_O_2_ (200 *μ*M) as a model control group. After 6 h, pictures were captured in five random fields. The tube formation assay was analyzed using the software of ImageJ. The number of tube-like structures and total branch lengths per field were counted.

### 2.9. Measurement of Reactive Oxygen Species (ROS)

2′,7′-Dichlorodihydrofluorescein diacetate (DCFH-DA) is a fluorescent indicator of H_2_O_2_ or other ROS formation used as a marker of oxidative stress in cells. HUVECs were plated and treated as described in Section 2.6. Then, cells were washed with PBS and incubated with 1% 1640 medium with 5 *μ*M DCFH-DA in the dark at 37°C for another 0.5 h. Subsequently, cells were washed three times with precooled PBS to remove DCFH-DA that failed to enter cells. Images were taken under a fluorescence microscope (Leica DMI3000 B, Germany). The green fluorescence intensity of each group was analyzed to quantify ROS production by Image-Pro Plus 6.0.

### 2.10. LDH, MDA, SOD, and NO Analysis

HUVECs were plated and treated as described in Section 2.6. Then, cells in each group were digested and collected with PBS (0.01 M, pH 7.4), and the cell homogenate was obtained by ultrasonic crushing. The activities of LDH, SOD, and NO in cell homogenate were detected according to the instructions on the corresponding kits. The absorbance of LDH, MDA, SOD, and NO was measured at 450 nm, 532 nm, 550 nm, and 550 nm following the manufacturer's instructions. Each experiment was performed in triplicate.

### 2.11. Total RNA Extraction, Reverse Transcription, and RT-qPCR Analysis

Total RNA was extracted by the Cell Total RNA Isolation Kit according to the manufacturer's instructions. Cells were treated as described in Section 2.6. The optical density (OD) at 260/280 nm was measured for RNA purity detection with the Nucleic Acid/Protein Analyzer and then converted to single-strand cDNA with a cDNA Synthesis System for RT-qPCR (abm). The RT-qPCR reaction conditions were set as follows: 95°C for 10 min, 40 cycles of 95°C for 15 s, and 60°C for 30 s. For RT-qPCR reactions, three independent biological samples were used for each experiment. The relative mRNA expression levels were calculated by the 2-*^ΔΔ^*CT method. All primers used in RT-qPCR were designed using the Prime-BLAST (NCBI) and synthesized in TSINGKE Biological Technology (Chengdu, China). The gene primer sequences are listed in Table 1.

### 2.12. Western Blot Assay

Cells were treated as described in Section 2.6. The HUVECs were washed twice with ice-cold PBS and then lysed with RIPA lysis buffer (RIPA lysis buffer : PMSF : protein phosphatase inhibitor : protein mixing enzyme inhibitor = 100 : 1 : 1 : 1) at 4°C. The protein concentration of each sample was quantified using the BCA protein assay kit according to the manufacturer's instructions. Then, adjust the protein concentration to the same by the lysis buffer. Protein loading buffer was added (total protein : loading buffer = 4 : 1) and heated for 5 min at 100°C. Then, equal amounts of protein from each group were loaded onto 10% SDS-PAGE and transferred to PVDF membranes. Then, the membranes were blocked with 5% skimmed milk for 2 h at room temperature followed by incubation overnight at 4°C with a primary antibody (GAPDH, *β*-actin, PI3K, Akt, eNOS, phospho-PI3K, phospho-Akt and phospho-eNOS, Bax, Bcl2, and caspase3) at a dilution of 1 : 1000. Subsequently, the membranes were washed three times with TBST and incubated with the appropriate HRP-conjugated goat anti-rabbit IgG (1 : 10000) for 2 h at room temperature. The protein band was detected by the ECL kit and quantified by ImageJ. GAPDH and *β*-actin were used as a standard reference. The relative density of each protein band was normalized to GAPDH or *β*-actin.

## 3. Data Analysis

All statistical analyses were performed using GraphPad Prism Version 8.00 (GraphPad Software, Inc.). Values were presented as the mean ± S.D. The differences were analyzed by a *t*-test when there were only two groups or assessed by one-way ANOVA when there were more than two groups. The *P* value (*P* < 0.05) was considered statistically significant among all the analyses.

## 4. Results

### 4.1. LEO Promoted Cell Proliferation and Inhibited H_2_O_2_-Induced Injury in HUVECs

**Figure 2:**
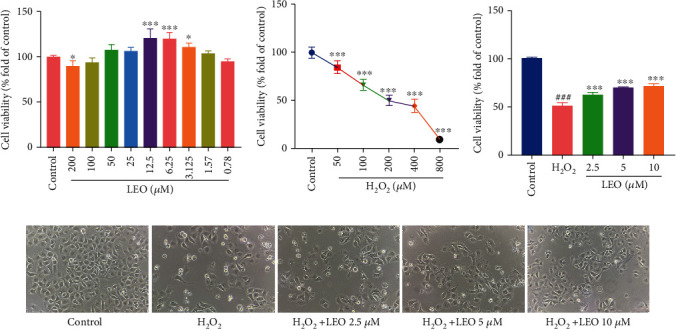
Detection of cell survival rate by MTT assay: (a) effect of LEO on the proliferation of HUVEC; (b) effect of H_2_O_2_ on the viability of HUVEC; (c) the survival rate of H_2_O_2_-induced injury following treatment with LEO at different concentrations; (d) effect of LEO on cell morphology after H_2_O_2_-induced injury in HUVECs. Values are presented as means ± S.D. (*n* = 6). ^#^*P* < 0.05, ^##^*P* < 0.01, and ^###^*P* < 0.001 vs. control group; ^∗^*P* < 0.05, ^∗∗^*P* < 0.01, and ^∗∗∗^*P* < 0.001 vs. H_2_O_2_ group.

The results showed that there was no significant change in cell morphology and no apparent cytotoxicity when treated with LEO at the concentration range of 0.78 *μΜ*-100 *μΜ* for 24 h compared with the control group. LEO at the concentration range of 3.125 *μΜ* to 12.5 *μΜ* showed a substantial effect on promoting cell proliferation in a dose-dependent manner, and the ability to promote cell proliferation was strongest at the concentration of 12.5 *μ*M. In contrast, when the concentration of LEO reached 200 *μ*M, it could significantly inhibit the proliferation of cells (*P* < 0.01), as shown in Figure 2(a). Therefore, 2.5 *μ*M, 5 *μ*M, and 10 *μ*M were selected as the optimal concentration for the study.

The results of the study on the role of H_2_O_2_ in inducing HUVEC injury showed that treatment with 50-1200 *μ*M H_2_O_2_ for 24 h decreased the survival rate of HUVECs in a concentration-dependent manner. 200 *μ*M H_2_O_2_ reduced the survival rate of HUVECs to about 50% (*P* < 0.001), as shown in Figure 2(b). The reduction degree is moderate, and the reproducibility was stable. Based on these results, 200 *μ*M H_2_O_2_ was selected as the moulding concentration for subsequent experiments to induce HUVEC oxidative injury.

To assess the protective effect of LEO on H_2_O_2_-induced injury, HUVECs were exposed to H_2_O_2_ (200 *μ*M) and LEO (2.5, 5, and 10 *μ*M) for 24 h. As shown in Figure 2(d), the cells in the control group adhered well with clear and smooth edges, while those in the H_2_O_2_ group were wrinkled into a star shape, and a large number of suspended cells and cell fragments were visible. Interestingly, there was a significant recovery in cell morphology, with little suspended cells and cell fragments after treatment with LEO. The cell survival rate was significantly increased in the treatment of the LEO group (2.5, 5, and 10 *μ*M) compared with the H_2_O_2_ group (*P* < 0.001) as shown in Figure 2(c).

### 4.2. LEO Promoted HUVEC Migration

**Figure 3:**
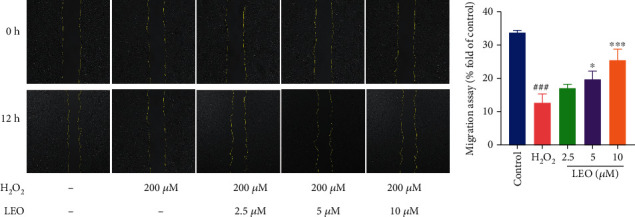
Detection of cell migration rate and wound closure. Values are presented as means ± S.D. (*n* = 3). ^#^*P* < 0.05, ^##^*P* < 0.01, and ^###^*P* < 0.001 vs. control group; ^∗^*P* < 0.05, ^∗∗^*P* < 0.01, and ^∗∗∗^*P* < 0.001 vs. H_2_O_2_ group. The bar chart shows quantitative data for HUVEC migration with the treatment of different concentrations of LEO.

Cell migration plays an important role in the growth and development of cells. To evaluate the effect of LEO on endothelial cell wound healing, we measured the area between the wound edges. As shown in Figure 3, H_2_O_2_ can significantly inhibit the migration of endothelial cells. At a lower concentration (2.5 *μ*M), LEO caused a slight wound closure (29.86 ± 1.28%) compared with the model group (25.55 ± 3.46%) at 12 h. In comparison, treatment with LEO at the concentration of 5 *μ*M and 10 *μ*M can extensively reduce wound width (34.38 ± 2.82%, 39.54 ± 2.23%) at 12 h in a dose-dependent manner.

### 4.3. LEO Enhanced Tube Formation and Rescued HUVEC Tube Injury

**Figure 4:**
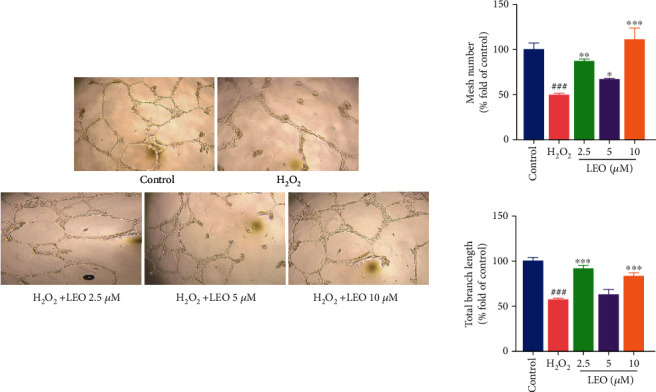
Evaluation for the tube formation in HUVECs. Images for the *in vitro* formed tubes in HUVECs. Values are presented as means ± S.D. (*n* = 3). ^#^*P* < 0.05, ^##^*P* < 0.01, and ^###^*P* < 0.001 vs. control group; ^∗^*P* < 0.05, ^∗∗^*P* < 0.01, and ^∗∗∗^*P* < 0.001 vs. H_2_O_2_ group. The bar chart shows quantitative data for HUVEC tube formation with the treatment of different concentrations of LEO.

Matrigel is a reasonable proxy for *in vivo* tube formation. The ability of endothelial cells to remodel and align is a requirement for the formation of new blood vessels during angiogenesis, and it can be tested by the in vitro tube formation assay. In the tube formation assay, LEO enhanced HUVEC tube-like structure formation after a 6 h incubation of HUVECs. As shown in Figure 4, H_2_O_2_ (200 *μ*M) significantly inhibited the formation of tube-like structures, while treatment with different concentrations of LEO enhanced HUVEC tube-like structure formation compared with the H_2_O_2_ group (200 *μ*M).

### 4.4. LEO Suppressed ROS Production Induced by H_2_O_2_

**Figure 5:**
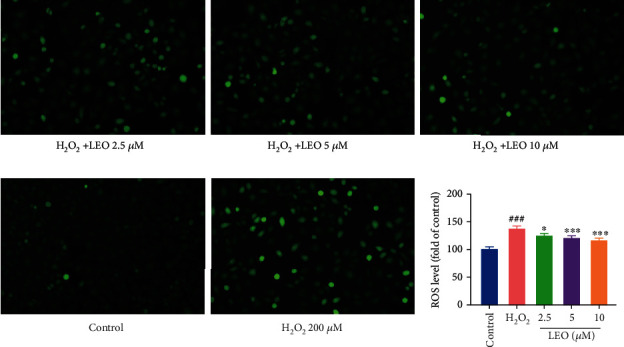
LEO inhibits oxidative damages in H_2_O_2_ stimulated in HUVECs. ROS generation in HUVECs was determined by measuring DCFH fluorescence. The ROS fluorescence intensity index was presented as the percentage of the control group. Data are represented as the mean ± S.D. of three separate experiments (*n* = 6). ^#^*P* < 0.05, ^##^*P* < 0.01, and ^###^*P* < 0.001 vs. control group; ^∗^*P* < 0.05, ^∗∗^*P* < 0.01, and ^∗∗∗^*P* < 0.001 vs. H_2_O_2_ group.

To investigate the effect of LEO on antioxidation, the intracellular ROS generation was evaluated by the DCFH-DA assay. As shown in Figure 5, results indicated that the signal intensity of DCFH-DA staining increased significantly compared with the control group (*P* < 0.001) after treatment with H_2_O_2_, which means that H_2_O_2_ can induce oxidative stress injury in HUVECs. While treat with LEO significantly reduced the level of excessive production of ROS induced by H_2_O_2_. ROS intensity in the H_2_O_2_ group was 136.01 ± 6.19, and it was reduced to 124.47 ± 3.26, 118.98 ± 5.12, and 116.39 ± 6.71 when treated with LEO (2.5, 5, and 10 *μ*M), respectively. These results suggested that LEO significantly reduced ROS overproduction in HUVECs after H_2_O_2_ induced oxidative stress in a dose-dependent manner.

### 4.5. LEO Regulated NO, LDH, MDA, and SOD in H_2_O_2_-Induced HUVECs

**Figure 6:**
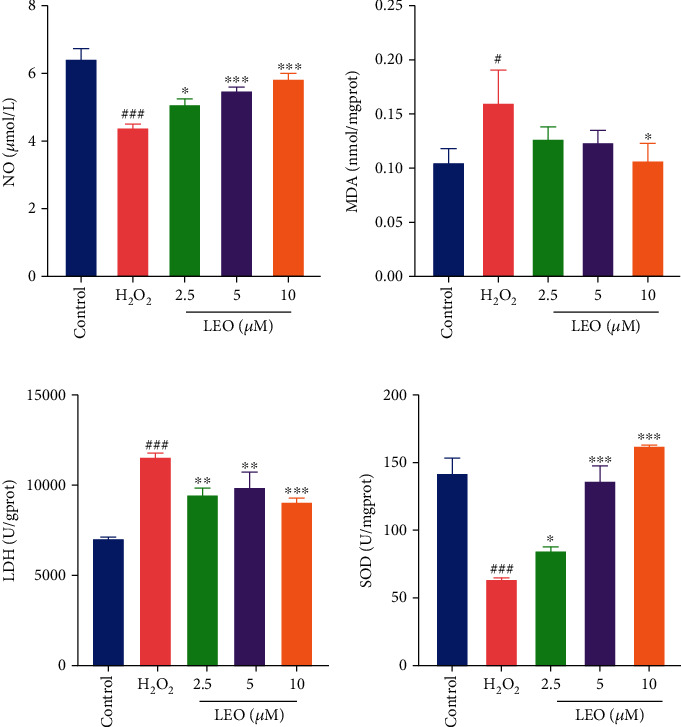
Effect of LEO on NO, MDA, LDH, and SOD levels in HUVECs treated with H_2_O_2_. Values are presented as means ± S.D. (*n* = 3). ^#^*P* < 0.05, ^##^*P* < 0.01, and ^###^*P* < 0.001 vs. control group; ^∗^*P* < 0.05, ^∗∗^*P* < 0.01, and ^∗∗∗^*P* < 0.001 vs. H_2_O_2_ group.

NO is an essential part of maintaining and improving the local blood flow and inhibiting thrombus formation. The decrease in its bioavailability is one of the important features of vascular endothelial cell injury. Malondialdehyde (MDA) production, lactate dehydrogenase (LDH), and superoxide dismutase (SOD) are biomarkers of oxidative stress. They reflect the damage degree of the cell's membrane function and integrity [25]. As shown in Figure 6, compared with the control group, the contents of LDH and MDA in cell homogenate were significantly increased (*P* < 0.001, *P* < 0.01), and the activity of NO and SOD was dramatically decreased in the H_2_O_2_ group (*P* < 0.001), indicating that the antioxidant capacity of HUVECs was unbalanced and significantly impaired. After treatment with LEO (2.5, 5, and 10 *μ*M), the contents of LDH and MDA were significantly decreased, and the activity of NO and SOD was significantly increased compared with the H_2_O_2_ group (*P* < 0.001, *P* < 0.001).

### 4.6. LEO Inhibited HUVEC Apoptosis Induced by H_2_O_2_ via Bax/Bcl2/Caspase3 Signaling Pathway

**Figure 7:**
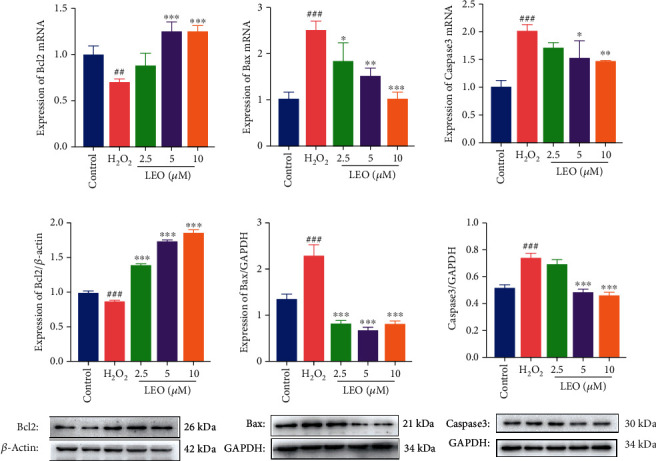
Effect of LEO on the mRNA expression and protein expression of Bax, Bcl2, and caspase3 in HUVECs: (a) effect of LEO on the mRNA expression of Bax, Bcl2, and caspase3 in HUVECs; (b) effect of LEO on the protein expression of Bax, Bcl2, and caspase3 in HUVECs. Values are presented as means ± S.D. (*n* = 3). ^#^*P* < 0.05, ^##^*P* < 0.01, and ^###^*P* < 0.001 vs. control group; ^∗^*P* < 0.05, ^∗∗^*P* < 0.01, and ^∗∗∗^*P* < 0.001 vs. H_2_O_2_ group.

To further explore the antiapoptosis effect of LEO, the expression of apoptosis-related genes and proteins was detected by RT-qPCR and western blot, respectively. As presented in Figures 7(a) and 7(b), LEO (2.5, 5, and 10 *μ*M) significantly suppressed the mRNA expression of Bax and caspase3 and improved the mRNA expression of Bcl2 compared with the H_2_O_2_ group (*P* < 0.05, *P* < 0.001). In addition, in the western blot assay, the same effect was shown on the protein expression of Bax, caspase3, and Bcl2, indicating that the mitochondrial apoptosis pathway was activated, while LEO could inhibit the apoptosis of HUVECs induced by H_2_O_2_.

### 4.7. LEO Regulated Oxidative Stress and Angiogenesis of HUVECs Induced by H_2_O_2_ via PI3K/Akt-eNOS Signaling Pathway

**Figure 8:**
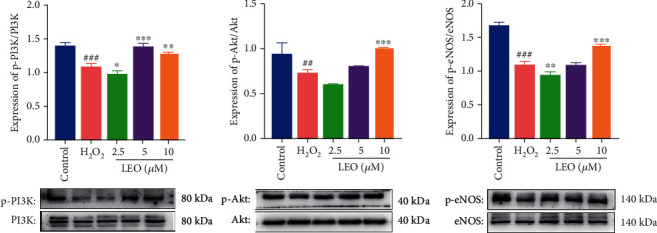
Effect of LEO on protein expression of PI3K, p-PI3K, Akt, p-Akt, eNOS, and p-eNOS in HUVEC. Values are presented as means ± S.D. (*n* = 3). ^#^*P* < 0.05, ^##^*P* < 0.01, and ^###^*P* < 0.001 vs. control group; ^∗^*P* < 0.05, ^∗∗^*P* < 0.01, and ^∗∗∗^*P* < 0.001 vs. H_2_O_2_ group.

Previous research suggested that the PI3K/Akt pathway was associated with cell proliferation, survival, metabolism, and finally regulating endothelial function. To investigate whether LEO can inhibit oxidative stress and promote angiogenesis through the PI3K/Akt signaling pathway, we further performed western blot assays. As shown in Figure 8, H_2_O_2_ downregulated phospho-PI3K, phospho-Akt, and its downstream target phospho-eNOS compared with the control group (*P* < 0.001), while those protein levels were all upregulated in a dose-dependent manner following the treatment of different concentrations of LEO (2.5, 5, and 10 *μ*M). Collectively, these results showed that LEO could regulate the apoptosis and hypoangiogenesis of HUVECs induced by H_2_O_2_ through activating the PI3K/Akt-eNOS pathway.

## 5. Discussion

Due to the characteristics of multitarget therapy, TCM has become a potential treatment for various diseases. At present, the antithrombotic treatment of TCM has been widely used in the clinic, especially in cardiovascular diseases. The significant efficacy of TCM in promoting blood circulation and removing blood clots has been recognized by the majority of patients, such as safflower injection, Danhong injection, and safflower yellow pigment injection.

Vascular endothelial cells can secrete vasoactive substances and regulate vascular function, and its injury will affect the normal proliferation, migration, and apoptosis of endothelial cells. Meanwhile, the injured endothelial cells can also cause plasma extravasation and angiogenesis disturbance and then lead to local circulation disturbance and thrombus [26]. Therefore, the repair of vascular endothelial injury can restore the homeostasis of vascular endothelial cells, thus improving the progress of thrombus-related diseases and restoring blood flow [27, 28] by promoting the formation of new blood vessels around the thrombus. Therefore, the repair of endothelial injury and promotion of angiogenesis can be used as indicators to evaluate the efficacy of drugs for promoting blood circulation and removing blood clots.

Oxidative stress is caused by the presence of ROS. A high level of ROS is one of the main factors causing oxidative stress and inducing endothelial nitric oxide (NO) biological activity damage [29, 30] and endothelial dysfunction and vascular remodeling [31–33] and finally leading to atherosclerosis, thrombosis, and other vascular-related diseases [34]. For a long time, the abnormality of ROS generation and the subsequent decrease of NO bioavailability in blood vessels are considered a copathogenic mechanism of endothelial dysfunction leading to various cardiovascular risk factors [35]. Researches indicated that long-term excessive ROS exposure might cause mitochondrial structure and function changes, which may induce endothelial dysfunction like senescence, apoptosis, and permeability changes and finally lead to thrombosis [36, 37]. Meanwhile, endothelial cell apoptosis can induce mitochondria apoptosis and stimulate ROS production, further aggravating endothelial cell damage, blocking microvascular circulation, and inducing cardiovascular embolic diseases, such as atherosclerosis and thrombosis [38–40]. Therefore, ROS is essential for vascular endothelial cell survival and cardiovascular function in health and disease [40, 41]; regulating oxidative stress and inhibiting cell apoptosis are effective treatments for thrombosis and cardiovascular diseases.

As one of the main forms of reactive oxygen species, H_2_O_2_ induces ROS production in different ways, resulting in typical vascular endothelial cell injury. Therefore, the H_2_O_2_-induced oxidative stress model is usually used to research drugs that are beneficial to blood vessels [42–44]. Our results showed that the activity of HUVECs was significantly decreased (*P* < 0.001), and the content of ROS was significantly increased (*P* < 0.001) in the model group (H_2_O_2_ treatment group), while treatment with different concentrations of LEO significantly improved the activity of HUVECs and downregulated the increase of ROS induced by H_2_O_2_ in a dose-dependent manner.

NO is known to have the strongest vasodilating effect, which can dilate blood vessels, improve microcirculation, regulate platelet activity, and promote angiogenesis and endothelial cell proliferation [45, 46]. Therefore, the decrease of its bioavailability is one of the important characteristics of vascular endothelial cell injury. Lactate dehydrogenase (LDH) and malondialdehyde production (MDA) are two stable enzymes in the cytoplasm and are rapidly released into the culture medium when the plasma membrane is damaged, so they were usually used as indicators of oxidative stress of cell membrane damage. The combined action of superoxide dismutase (SOD) and other endogenous antioxidants can effectively scavenge intracellular ROS and play an important role in preventing cellular damage caused by oxidative stress. In this study, in the H_2_O_2_ treatment group, the content of LDH and MDA increased significantly. The content of NO and SOD decreased particularly compared with the control group (*P* < 0.001), indicating that H_2_O_2_ treatment induced the imbalance of antioxidant capacity and HUVEC injury. However, treatment with LEO (2.5, 5, and 10 *μ*M) decreased the content of LDH and MDA and increased the NO content and the SOD activity significantly, indicating that LEO can improve the oxidative damage of HUVECs induced by H_2_O_2_.

The formation of new blood vessels is a complex process, which is a manifestation of the normal function of endothelial cell proliferation and migration. The formation of new blood vessels around the thrombus can restore the blood flow of some occlusive veins and relieve the thrombus symptoms. Therefore, the promotion of angiogenesis is a potential treatment for diseases such as acute myocardial infarction and ischemic heart failure [47]. Our results suggested that treatment with different concentrations of LEO significantly improved the ability of cell migration and tube formation (*P* < 0.001) induced by H_2_O_2_.

Previous studies have shown that the PI3K/Akt pathway positively activated Akt and eNOS in vascular endothelial cells. The activated phosphor-Akt not only inhibit the production of ROS [48] and promote the proliferation [45], migration [49], and angiogenesis [50–57] but also inhibit the proapoptotic Bcl2 family members such as Bad, Bax, and caspase3 [58–63] and improve vascular dysfunction at last [64]. What is more, activated Akt is conducive to promoting the phosphorylation of eNOS, stimulating endothelial cells to secrete a large amount of NO, thus maintaining and improving local blood flow [65, 66] and inhibiting thrombosis. These results indicated that sequences of beneficial physiological activities were mediated by PI3K/Akt/eNOS signaling pathways. Our results showed that H_2_O_2_ significantly promoted the expression of apoptosis factors such as Bax and caspase3 and inhibited the expression of antiapoptosis factor Bcl2 and the phosphorylation of PI3K, Akt, and eNOS proteins, while the intervention with different concentrations of LEO significantly upregulated the expression of the phosphorylated protein of PI3K, Akt, and eNOS in a dose-dependent manner. Those results suggested that LEO could successfully reverse the H_2_O_2_-induced cell injury by activating the PI3K/Akt-eNOS.

**Figure 9:**
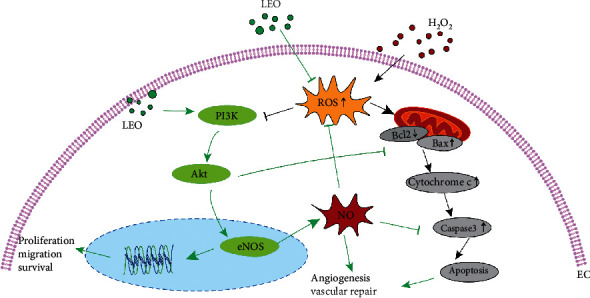
H_2_O_2_ can induce the expression of apoptotic protein Bax and inhibit the phosphorylation of PI3K/Akt by increasing the content of ROS, while LEO can promote the phosphorylation of PI3K/Akt and further promote the expression of eNOS, thus promoting the survival, proliferation, migration, and NO release of endothelial cells. At the same time, phosphorylated-Akt can also inhibit the expression of apoptotic proteins such as Bcl2 and Bax, thus inhibiting endothelial cell apoptosis induced by ROS. “←” indicates activation, and “⟝” indicates inhibition.

To sum up, our research indicated that LEO can efficiently reduce the production of ROS, repair vascular endothelial injury, and promote angiogenesis via the PI3K/Akt-eNOS pathway, as shown in Figure 9. Therefore, LEO might be a potential candidate in preventing oxidative stress-induced vascular-related diseases. These findings may provide an important scientific basis for further study of the effect of *Leonurus japonicus* Houtt on promoting blood circulation and removing blood clots.

## Figures and Tables

**Table 1:** The gene primer sequence used for RT-qPCR.

Gene	Forward (5′⟶3′)	Reverse (5′⟶3′)
PI3K	5′ACATGGCTCTGCAAGATGCT3′	5′GGAGGCATCTCGGACCAAAA3′
AKT	5′TCGGCAGGTGTCTTCTCAAT3′	5′ACCCATTGCCATACCACGAG3′
*eNOS*	5′GCCGGAACAGCACAAGAGTTA3′	5′CCCTGCACTGTCTGTGTTACT3′
*Bax*	5′TGAGCAGATCATGAAGACAGGG3′	5′TGAGACACTCGCTCAGCTTC3′
Bcl2	5′TCACTTGTGGCCCAGATAGG3′	5′GATAACGGAGGCTGGGATGC3′
Caspase3	5′TCCACAGCACCTGGTTATTATTCT3′	5′ATGGCACAAAGCGACTGGAT3′
GAPDH	5′GGATCTGACAGTCCGTCTTGAGAA3′	5′CCATTGAAGTCAGTGGACACAACC3′

## Data Availability

The underlying data of the study can be obtained by contacting the authors if it is reasonable.
